# The effectiveness of a simple ‘Code Red’ transfusion request policy initiated by pre-hospital physicians

**DOI:** 10.1186/1757-7241-20-S1-O1

**Published:** 2012-03-22

**Authors:** AE Weaver, J Thompson, DJ Lockey

**Affiliations:** 1 London’s Air Ambulance, Barts and the London NHS Trust, London, UK

## Background

Recent approaches to life threatening traumatic haemorrhage have emphasized the early use of blood products in treatment [1]. We have implemented a transfusion request policy where a pre-hospital physician can request the presence of a transfusion pack (consisting of 6 units PRBC, 4 units FFP) at the destination trauma centre.

## Aims

This study was initiated to establish whether the three simple criteria used to activate a ‘Code Red’ pre-hospital transfusion request (suspicion or evidence of active haemorrhage, systolic BP <90mmHg, failure to respond to fluid bolus) identified seriously injured patients and whether they actually received the requested blood.

## Methods

Data was collected prospectively on all ‘Code Red’ pre-hospital requests in a 30 month period brought to our Trauma Centre. Injury severity scores, mortality, mechanism of injury and use of blood products were recorded.

## Results

176 ‘Code Red’ activations were made in the 30 month period. Mechanism of injury was penetrating in 59 (33.5%), RTC related in 76 (43.2%), falls in 22 (12.5%) and ‘other’ in 19 (10.8%). 127 patients were received at our trauma centre and data was available in 92 patients. 29 (31.5%) died. Injury Severity Score (ISS) was 29.2 (mean) and 27 (median) overall. ISS was 22.9 (mean) and 25 (median) in survivors and 30 (mean) and 28 (median) in those that died. Records of blood product use revealed that overall a mean 11.6 units of PRBC and mean 7.2 units of FFP were administered. Patients that survived received a mean 7.8 PRBC and 6.0 FFP while those that died received a mean 18 PRBC and 9.7 FFP. 6 patients received no blood products. Of the 6, 5 were victims of stab wounds and all had a systolic BP>90mmHg on arrival in hospital.

## Conclusions

The use of simple pre-hospital criteria allows physicians to identify a group of trauma patients with severe injury and a requirement for significant transfusion.
